# An Interesting Case of Gangrenous Balanitis

**Published:** 1909-09

**Authors:** 


### An Interesting Case of Gangrenous Balanitis.

I. BY WILLIAM MEYER.

The following case deserves to be reported for several reasons. First, for the long duration of the affection; second, for its obstinate resistance to dozens of different remedies, and third, for its rapid, one might say, magical, yielding to another remedy when all hopes of its being influenced by purely medicinal treatment were about abandoned.

Mr. W. K., age 60, applied to me in October, 1908, for treatment for ulcerative balanitis. He gave the following history: His disease began in February, 1908. On the superior aspect of the glans penis appeared a spot resembling a mosquito bite, which itched intensely. He applied some vaselin, which relieved the itching somewhat, but the spot became converted into an ulcer with profuse secretion and intense itching. He then consulted Dr. M of Astoria, who made no diagnosis, but gave him a yellow powder, probably iodoform, which brought no relief. He then gave him a white powder, which aggravated the trouble, the ulcer increasing in size and the itching increasng in intensity. The doctor then gave up local treatment, giving him some medicine internally. He told him to keep the glans dry, but this was easier said than done, because there kept on secreting a yellowish, offensive liquid, the secretion being continued day and night.

He then consulted a Dr. K. of New York, who agreed to cure him for $15 in two weeks. He gave him lead and opium wash, a 2 per cent solution of permanganate of potassium, then internal treatment and several other local applications. None of them did any good. On the contrary, the ulceration kept on progressively both in surface and in depth.

When he applied to me for treatment a large part of the glans penis was ulcerated, the meatus also being invaded. He also complained at the time of a burning pain in urination which often made him abstain from urinating as long as possible.

I used on him a great number of different remedies, among which may be mentioned the following: lead and opium lotion, bismuth subgallate, ichthyol ointment, an ointment of silver nitrate from one-half to one per cent strength, balsam of Peru, pure, balsam of Peru, 8, and unguentum fuscum, 22. All the above had practically no effect, with the exception of the last ointment, which relieved him somewhat, but only temporarily.

In January, 1909, the patient had an attack of pneumonia, and during the time of his illness his local trouble was naturally neglected. When he recovered from his pneumonia I began to treat him again for his local trouble, which began to worry him and me very considerably. It was absolutely non-responsive to every kind of treatment. I used corrosive sublimate solution 1:5000, peroxide of hydrogen, argyrol, etc., but the case progressively getting worse, I sent him for consultation to Dr. William J. Robinson.

II. BY WM. J. ROBINSON.

The above referred to patient, W. K., came to my office on March 22, 1909, with a letter from Dr. Wm. Meyer, asking suggestions for treatment. The glans presented a mass of ulceration on both the superior and inferior aspects and also within the meatus. Ill-smelling pus was exuding in abundance. Retracting the prepuce was difficult and painful. A microscopic examination revealed numerous cocci, but the Unna-Ducrey bacillus could not be identified. Examination of the urine and the bladder revealed a mild cystitis.

I advised the application several times a day of a solution of copper sulphate 1:500, gradually increasing the strength to 1:100. The application was to be made several times a day for several minutes at a time, the glans being dusted in the intervals with such powders as airol, europhen, etc.

I did not see the patient again until April 28th. On that day I found his condition very much aggravated, the ulcerations had gone deeper, extending practically over the entire surface of the glans and also over more than an inch within the urethral canal. He had severe pain on urinating and also on walking. He demanded that something radical be done for it now, as he could not suffer any longer with it and suggested that the glans be amputated if there was no medicinal treatment that would cure it.

Having just previously cured two very obstinate cases of phagedenic ulceration, which had resisted all other treatment, by the use of chinosol, I decided to try this drug. I advised dipping the penis four times a day for fifteen minutes each time in a 1:1000 solution of chinosol, afterwards dusting the glans with pure chinosol.

The patient came three days later, namely, on May 1st, and on that day his entire demeanor as well as the picture of the disease had entirely changed. He was full of gratitude and profuse in thanks. He stated that the pain on urination disappeared within an hour or two after commencing the treatment. (I wish to remark in parentheses, that chinosol, besides its antiseptic properties, seems to possess a decidedly anesthetic effect in some cases.) The secretion rapidly diminished and there were healthy granulations visible throughout.

To make this report as brief as possible: within one week the entire glans was healed, not a trace of any ulceration remaining. It is also rather remarkable that the ulcerations healed smoothly without leaving any cicatrices, and it is impossible to tell at the present time that the glans had been the seat of deep ulcerations.

Since then I treated three other cases of extensive phagedenic ulceration and chancroids with chinosol solution and chinosol powder and the results have been in the highest degree satisfactory in every case. In some cases pure chinosol seems to be too strong and I then use it diluted with boric acid or talcum, in strengths of 5 to 20 per cent.—*Critic and Guide,* August, 1909.

